# Construction and optimization of macromolecular structure model of Tiebei lignite

**DOI:** 10.1371/journal.pone.0289328

**Published:** 2023-08-07

**Authors:** Jinzhang Jia, Lingyi Xiao, Dongming Wang, Dan Zhao, Yinghuan Xing, Yumo Wu

**Affiliations:** 1 College of Safety Science and Engineering, Liaoning Technical University, Fuxin, Liaoning, China; 2 Ministry of Education, Key Laboratory of Mine Thermal Power Disaster and Prevention, Fuxin, Liaoning, China; 3 Faculty of Civil Engineering and Architecture, Zhanjiang University of Science and Technology, Zhanjiang, Guangdong, China; Monash University Malaysia, MALAYSIA

## Abstract

Mastering the molecular structure of coal is important for the effective utilization of coal. For a detailed study of the microstructural characteristics of Tiebei lignite, its molecular structure was characterized by elemental analysis, solid ^13^C nuclear magnetic resonance (^13^C NMR), Fourier-transform infrared (FT-IR) spectroscopy, X-ray photoelectron spectroscopy (XPS), and X-ray diffraction (XRD). The results showed that the aromatic carbon content of Tiebei lignite was 51.98%, the aromatic carbon structure was mainly composed of benzene and naphthalene, and the ratio of aromatic bridgehead carbon to surrounding carbon X_bp_ was 0.14. Oxygen existed in phenol, ether, carbonyl, and carboxyl; nitrogen-containing structures mainly existed in the form of pyrrole and pyridine; sulfur mainly existed in thiophene sulfur; and aromatic substitution was mainly in the form of trisubstitution. The molecular formula of the macromolecular structure model of Tiebei lignite was C_190_H_161_O_57_N_2_, and the ^13^C NMR spectrum of the model was in good agreement with the experimental results, which fully verified the accuracy of the macromolecular structure model of Tiebei lignite. The construction of a macromolecular structure model of Tiebei lignite is essential to intuitively understand the molecular structure characteristics of Tiebei lignite and to provide theoretical support and guidance for the micromechanism research and prevention of lignite spontaneous combustion and other disasters.

## 1. Introduction

Coal is a kind of abundant fossil energy distributed around the world that plays an important role in the fields of global industry, fuel, and energy [1]. In recent years, with the consumption of high-quality coal resources, the development and utilization of lignite have become effective ways to alleviate the energy shortage. The rich active groups and developed pore structure of lignite, however, have created a high tendency for spontaneous combustion, which has significantly limited the industrial utilization of lignite [2, 3]. The spontaneous combustion mechanism of coal is complex [4, 5], and the physical and chemical interactions may occur at the same time, making it difficult to distinguish the experiments. Therefore, understanding the macromolecular structure of lignite from the microscopic point of view holds guiding significance for the development and utilization of lignite, and fully understanding the molecular structure characteristics of lignite provides the breakthrough point for developing modern coal chemical industry.

In 1942, Fuchs established a lignite model. After continuous improvement and development, the coal macromolecular structure model has evolved through the Fuchs model [6], Given model [7], Wiser model [8], and Shinn model [9]. The Fuchs model was the first coal molecular model proposed, but it cannot characterize the arrangement and connection mode of the carbon skeleton structure. The Given model was the first model to construct coal molecules based on instrumental analysis, which identified the condensed aromatic structure in coal, but it did not consider the structure of sulfur atoms and ether bonds. The Wiser model pioneered the construction of sulfur atoms in the coal molecular structure model, and although it can reflect the basic structural characteristics of coal molecules, it is not well considered in the three-dimensional spatial chemical structure. At present, the Shinn coal molecular model is recognized by most scholars and contains heteroatom structures, such as sulfur and nitrogen, but it does not consider the existence of small molecular compounds in the coal structure. With the development of numerical simulation and computer technology, the development of coal molecular structure model has advanced significantly. Lian et al. [10] used X-ray photoelectron spectroscopy (XPS) and ^13^C nuclear magnetic resonance (^13^C NMR) experiments to analyze and construct the coal molecular model and clarified the mechanism of Dense medium component scaffold (DMC-S) aggregation. Zhang et al. [11] used elemental analysis, high-resolution transmission electron microscopy (HRTEM), XPS, and other techniques to construct the molecular structure of bituminous coal, to further understand the relationship between the structure and behavior of coal at the molecular level. Marcano et al. [12] determined that the coal structure consists of a covalent bond network of aromatic clusters with extensive hydrogen bonds through XPS and small-angle neutron scattering (SANS) experimental analysis. Cui et al. [13] analyzed 12 characteristic parameters of anthracite’s carbon skeleton structure and coal structure using ^13^C NMR spectrum, attenuated total reflection Fourier-transform infrared (FT-IR) spectrum, and quantitative chemical analysis; built a molecular structure model of anthracite; and calculated its aromaticity, hydrogen aromaticity, and average aromatic nucleus size. Mokone [14] used petrographic analysis, elemental analysis, ^13^C NMR, X-ray diffraction (XRD), and HRTEM to characterize the experiment and used the experimental data to construct the molecular structure of polycyclic aromatic hydrocarbons with oxygen, nitrogen, and sulfur, which provided a better and more accurate measurement of aromaticity and bone density.

Although many scholars have conducted a lot of research work and have made some progress, the problem of coal structure still has not been solved because of its complexity, diversity, amorphousness, and heterogeneity. Lignite is highly complex and heterogeneous, and research on its molecular structure is still in the exploratory stage [15]. A large number of testing techniques such as solid ^13^C nuclear magnetic resonance (^13^C NMR), X-ray diffraction (XRD), X-ray photoelectron spectroscopy (XPS), Raman spectroscopy (Raman) and Fourier-transform infrared spectroscopy (FT-IR) can be used to study the structural characteristics of coal. Computer-aided molecular design technology is widely used in the study of coal macromolecular structure model construction. Therefore, we selected Tiebei lignite as the research object. The chemical parameters of the macromolecular structure of Tiebei lignite were qualitatively and quantitatively characterized by elemental analysis, FT-IR analysis, XPS, XRD analysis, and ^13^C NMR analysis. We constructed an initial plane model structure of Tiebei lignite using KingDraw software and then constructed the structural model of Tiebei lignite with the lowest macromolecular energy using Materials Studio software. We optimized the structural model of coal macromolecules to construct the Tiebei lignite macromolecular model. We verified the rationality of the model by comparing the experimental spectrum of ^13^C NMR with the simulation diagram. The results of this study reveal the microscopic mechanism of Tiebei lignite at the molecular level and provide important theoretical support for the prevention and control of coal spontaneous combustion, coal and gas outburst, and other disasters.

## 2. Materials and methods

### 2.1 Coal sample preparation

We selected the lignite from the Tiebei Coal Mine in the Inner Mongolia Autonomous Region of China as the research object. The preparation of coal samples strictly followed the provisions of the Preparation Method of Coal Samples (GB474-2008). With lignite in an air-dried state as raw coal, the coal samples were repeatedly crushed, screened, and shrunk by a crusher and vibrating screen machine, and the coal samples were screened into 200 meshes for sealed preservation. We used an Elementar Vario EL to determine the contents of C, H, N, and S in the coal samples. The elemental analysis results of Tiebei lignite coal samples are presented in Table 1.

**Table 1:** Elemental analysis of lignite.

Coal sample	Elemental analysis, w_daf_ (%)				
	C	H	O	N	S
Lignite	58.93	4.16	23.55	0.79	0.11

### 2.2 ^13^C NMR analysis

^13^C NMR spectrum was measured by using a Bruker Avance NEO 400WB solid-state spectrometer in Bruker, Germany. When the resonance frequency was 100.66 MHz, the magnetic field intensity is 9.4T, the standard sample is adamantane, the sampling time was 0.026 s, the cycle delay time was 0.5 s, and the magic angle speed was kept at 8 kHz.

### 2.3 FT-IR analysis

In this study, we used Thermo Scientific Nicolet iS20 FT-IR spectrometer for FT-IR measurement. We mixed a 200-mesh coal sample with potassium bromide powder at a mass ratio of 1:200, and then, we fully ground it to make a transparent sheet with a thickness of 0.2–0.5 mm. The test wave number was 400–4000 cm-1, the resolution was 4 cm-1, and the number of scans was 32.

### 2.4 XPS analysis

We used Thermo Scientific K-Alpha X-ray photoelectron spectrometer and pressed a proper amount of the coal sample on the sample tray and put into the sample room of the XPS instrument. When the pressure in the sample room was less than 2.0×10–7 mbar, we sent the coal sample to the analysis room with a spot size of 400 μm, a working voltage of 12 kV, and a filament current of 6 mA. The full-spectrum scanning pass energy was 150 eV, and the step size was 1 eV.

### 2.5 XRD analysis

We used Rigaku Smart Lab SE X-ray diffractometer with a test range of 5–90, a scanning speed of 2°/min, a tube voltage of 40 kV, a current of 40 mA, and Cu-Kα ray as the light source.

### 2.6 Model construction and optimization

According to the analysis data of aromatic structure, fatty carbon structure, and heteroatom structure [16] of Tiebei lignite, we deduced the molecular formula of Tiebei lignite. The macromolecular plane structure model of Tiebei lignite was introduced into the molecular simulation software Materials Studio by using KingDraw software. After it was saturated by hydrogenation, the macromolecular plane structure model of Tiebei lignite was simply and preliminarily optimized by using Clean tool, until the macromolecular structure model of Tiebei lignite remained unchanged and remained stable. Geometric optimization of molecular mechanics and annealing optimization of molecular dynamics are carried out on the macromolecular structure model of Tiebei lignite through Forcite module. The specific parameters are that the maximum number of iteration steps is 3000, annealing treatment and dynamics treatment, COMPASS force field is selected, the calculation accuracy is Fine, the Charges term is set to Forcefield assigned, and NVT dynamics ensemble (300K‒600K, 5 cycles), and finally a stable three-dimensional molecular structure model of Tiebei lignite with the lowest energy is obtained. Finally, we compared the ^13^C NMR spectrum of the model with the experimental ^13^C NMR spectrum to verify the accuracy of the model [17].

## 3. Results and discussion

### 3.1 ^13^C NMR analysis

**Fig 1:**
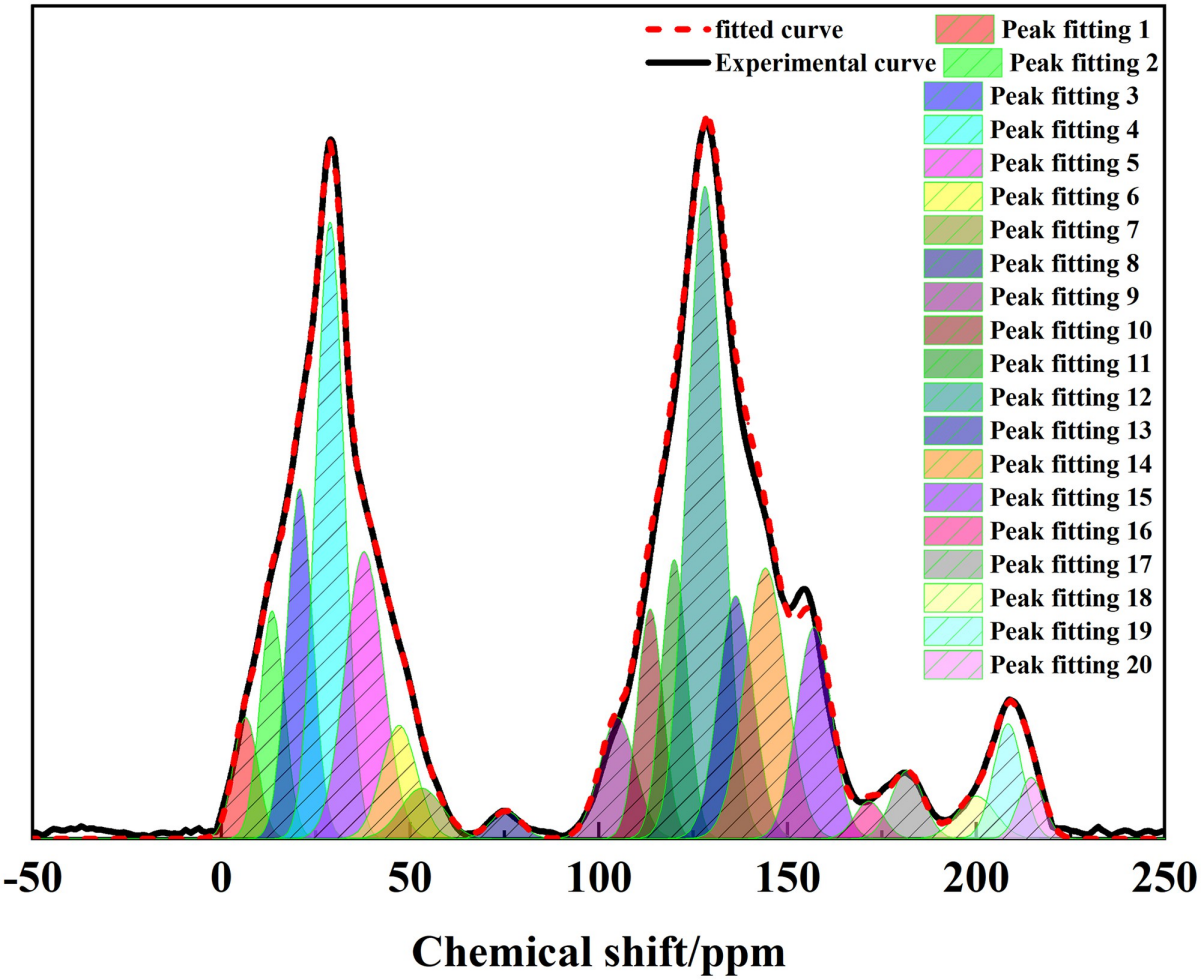
Fitting spectrum of ^13^C NMR peaks of lignite.

We fitted the ^13^C NMR experimental results of Tiebei lignite using Origin software. As shown in Fig 1, we divided the ^13^C NMR peak fitting curve of Tiebei lignite into 20 peaks, corresponding to different functional groups and contents of carbon in the molecular structure [18]. We then divided the peaks into three chemical regions: fatty carbon (0–90 ppm), aromatic carbon (90–170 ppm), and carboxyl/carbonyl carbon (170–220 ppm), with the highest proportion of aromatic carbon being 51.98%. Fatty carbon accounted for 40.63%, and the carboxyl/carbonyl carbon ratio was the smallest, at only 7.49%. Table 2 shows the parameters of the carbon-containing functional groups in the ^13^C NMR spectrum of lignite and the ^13^C NMR peak position and chemical shift attribution referred to in the related literature [19–21].

**Table 2:** Parameters of the functional groups containing carbon in ^13^C NMR of lignite.

Peak number	Chemical shift (ppm)	Proportion (%)	Functional group
1	6.41	2.28	Lipid methyl carbon *f*_al_^*^
2	13.45	4.30	Lipid methyl carbon *f*_al_^*^
3	20.76	6.97	Aromatic methyl carbon f_al_^*^
4	28.77	13.27	Methylene carbon f_al_^H^
5	37.82	8.45	Methylene carbon f_al_^H^
6	47.12	3.06	Methylene carbon f_al_^H^
7	53.11	1.64	Oxygen-containing fatty carbon f_al_^O^
8	75.43	0.66	Oxygen-containing fatty carbon f_al_^O^
9	104.89	3.42	Protonated aromatic carbon f_a_^H^
10	113.56	4.51	Protonated aromatic carbon f_a_^H^
11	119.93	5.48	Protonated aromatic carbon f_a_^H^
12	128.09	17.67	Protonated aromatic carbon f_a_^H^
13	136.24	6.55	Bridging aromatic carbon f_a_^B^
14	144.12	8.64	Alkylated aromatic carbon f_a_^S^
15	157.01	5.71	Oxygen aromatic carbon f_a_^P^
16	171.53	0.83	Carboxyl f_a_^C^
17	181.72	1.67	Carboxyl f_a_^C^
18	200.01	1.25	Carbonyl f_a_^C^
19	208.43	2.54	Carbonyl f_a_^C^
20	214.58	1.20	Carbonyl f_a_^C^

As shown in Table 2, the fatty carbon in Tiebei lignite was mostly methylene and methylene carbon. It was evident that the alkyl side chains in Tiebei lignite structure were mainly long chains and cycloalkanes. Through calculation, we obtained the structural parameters of Tiebei lignite, as shown in Table 3: protonated aromatic carbon (f_a_^H^) accounted for 31.08%, bridged aromatic carbon (f_a_^B^) accounted for 6.55%, lateral aromatic carbon (f_a_^S^) accounted for 8.64%, oxygen substituted aromatic carbon (f_a_^P^) accounted for 5.71%, and nonprotonated aromatic carbon (f_a_^N^) accounted for 20.90%. The aromatic carbon ratio (f_a_^’^) was 51.98%, and the ratio of the bridging carbon to peripheral carbon (X_bp_) in the coal macromolecular structure characterized the polycondensation degree of aromatic structure in the structure. The polycondensation degree of the aromatic structure of Tiebei lignite was 0.14. The calculation formula is as follows:

$$X_{bp}=\frac{f_{a}^{B}}{f_{a}^{H}+f_{a}^{P}+f_{a}^{S}}.$$ (1)

**Table 3:** ^13^C NMR structural parameters of lignite.

Coal sample	*f*_al_^*^	*f*_al_^H^	*f*_al_^O^	*f*_a_^H^	*f*_a_^B^	*f*_a_^S^	*f*_a_^P^	*f*_a_^N^	*f*_a_^C^	*f*_al_	*f*_a_	*f*_a_^’^
Lignite	13.55	24.78	2.30	31.08	6.55	8.64	5.71	20.90	7.49	40.63	59.47	51.98

These parameters provide an important basis for constructing the macromolecular structure model of Tiebei lignite.

### 3.2 FT-IR analysis

**Fig 2:**
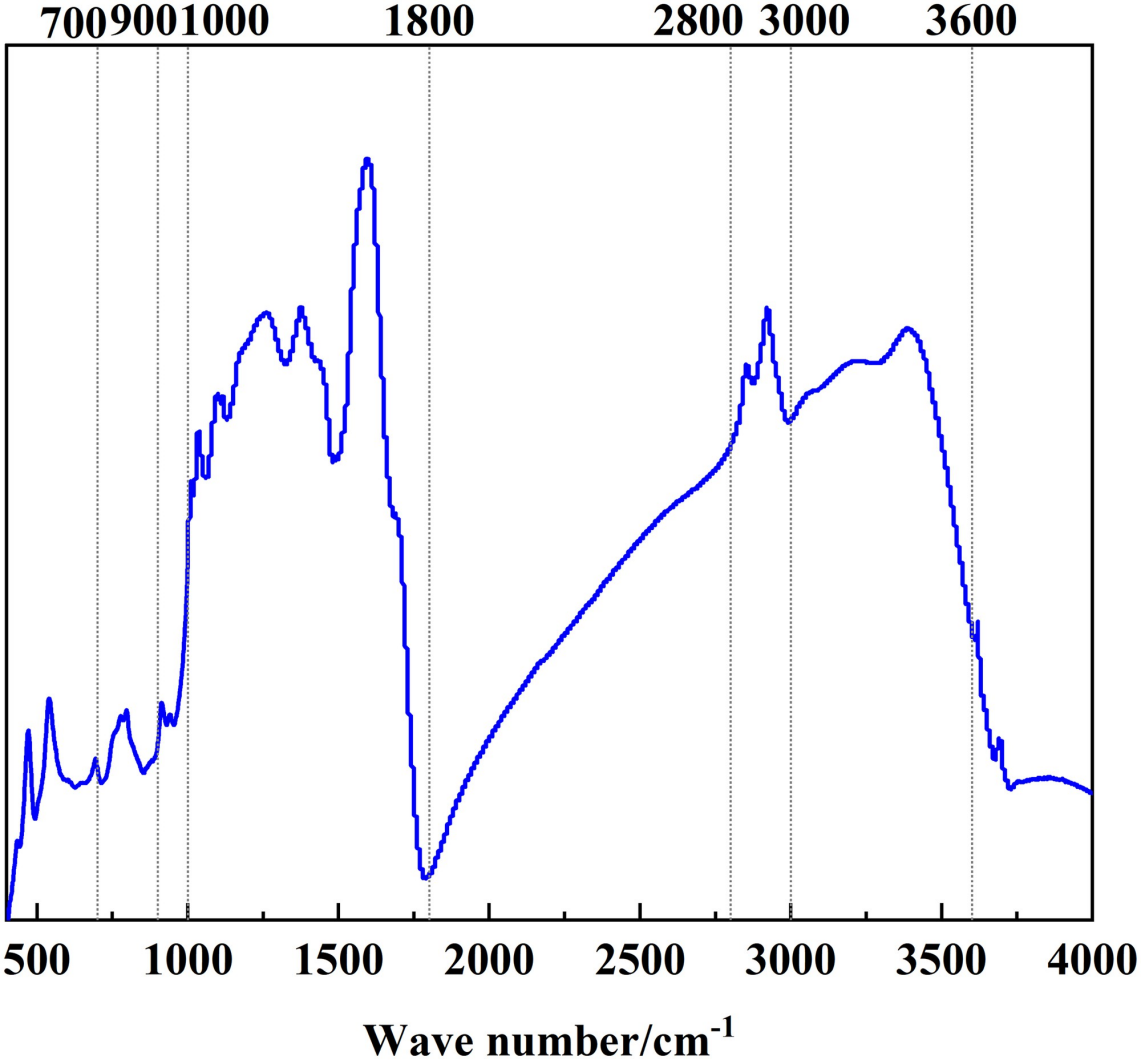
FT-IR spectrum of lignite.

**Fig 3:**
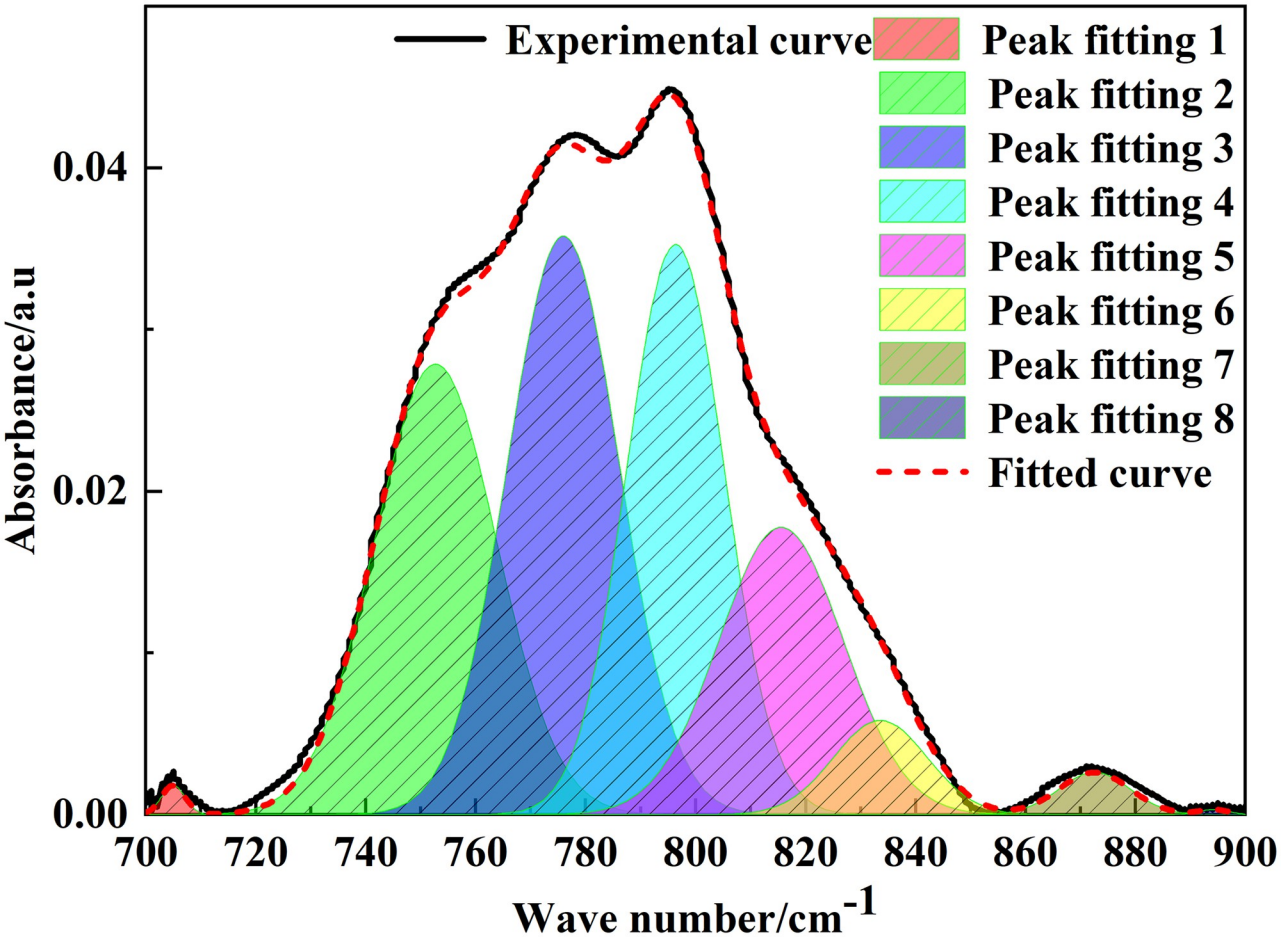
Infrared spectrum fitting of lignite in 700–900 cm^-1^ band.

**Fig 4:**
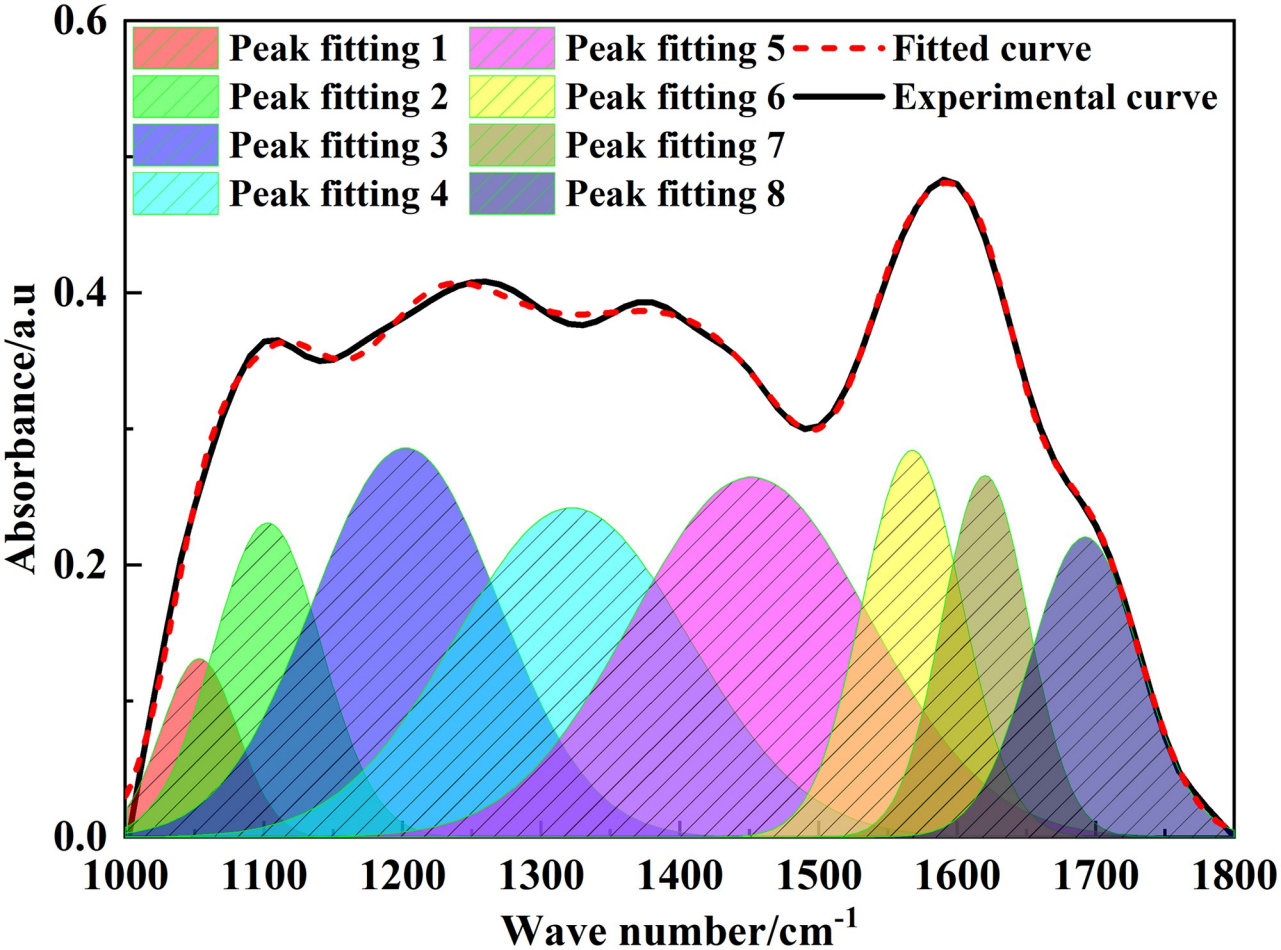
Infrared spectrum fitting of lignite in 1000–1800 cm^-1^ band.

**Fig 5:**
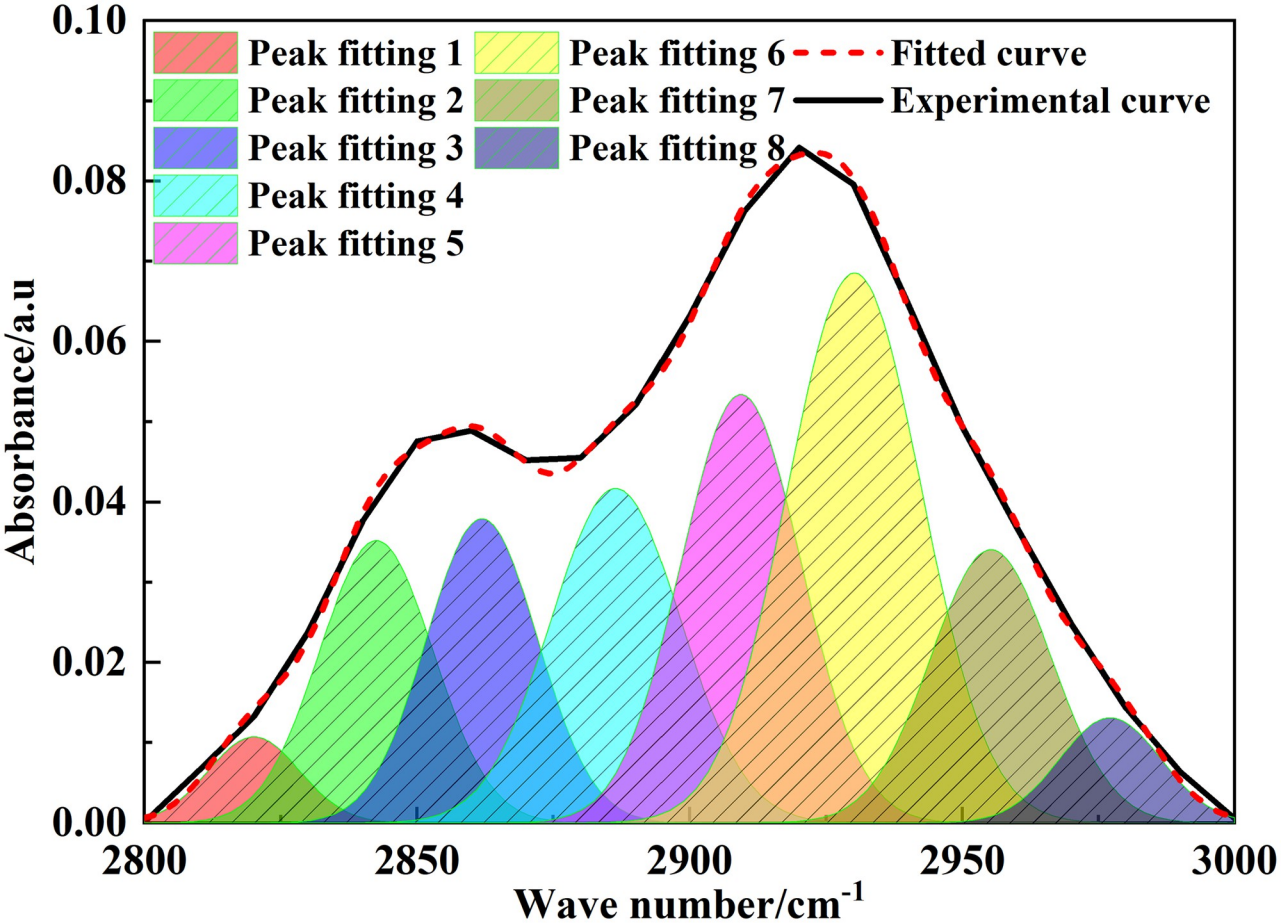
Infrared spectrum fitting of lignite in 2800–3000 cm^-1^ band.

**Fig 6:**
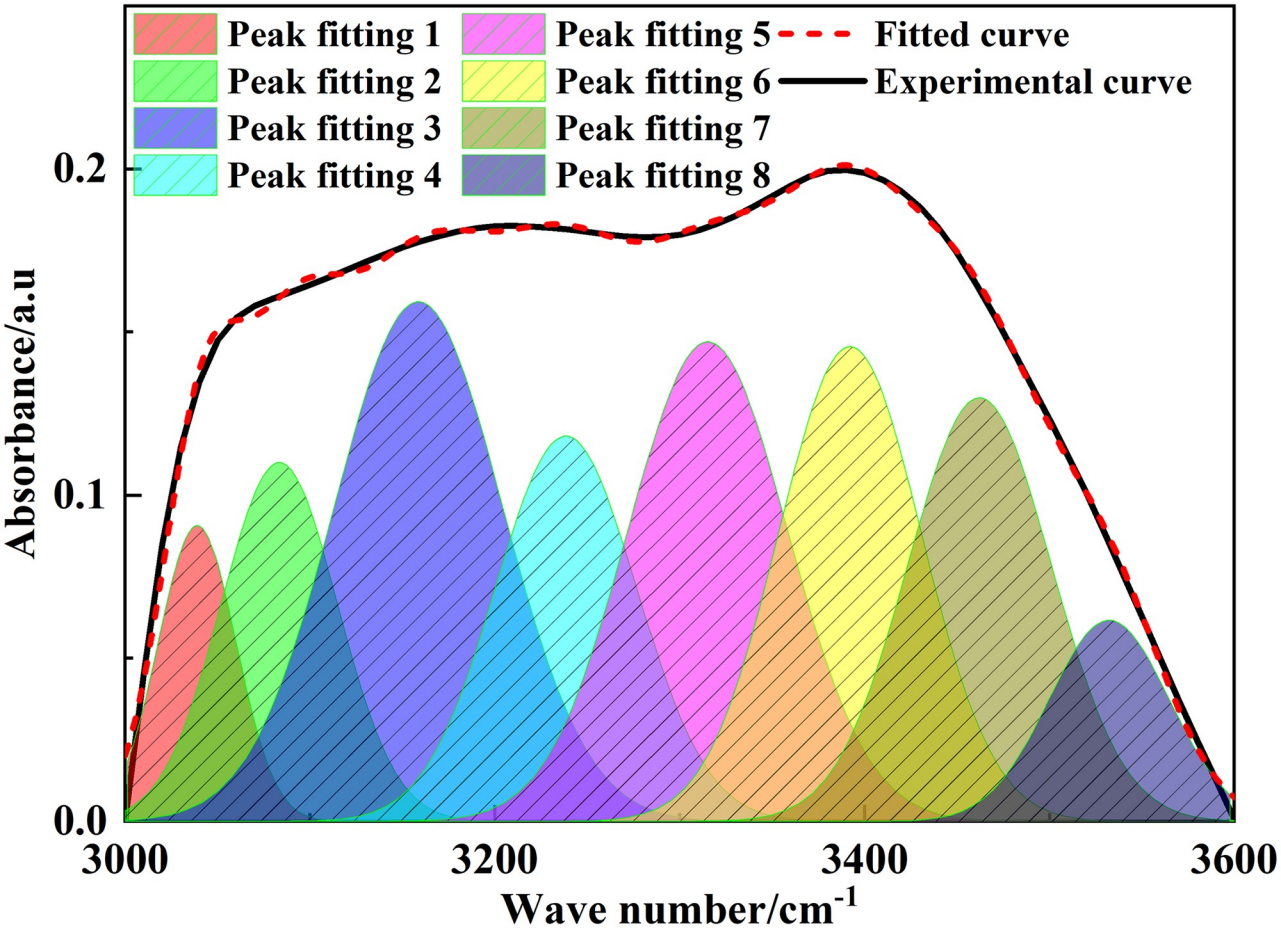
Infrared spectrum fitting of lignite in 3000–3600 cm^-1^ band.

The position and intensity of infrared absorption peaks are related to the molecular composition and content of functional groups [22]. Fig 2 shows the FT-IR spectrum of Tiebei lignite. Fig 3–6 shows the FT-IR fitting spectrum of Tiebei lignite. We fitted the infrared spectrum experimental data of Tiebei lignite using Origin software, and the functional group attribution is shown in Tables 4–7. We divided the FT-IR spectrum into four absorption bands: the 700–900 cm^-1^ band was an aromatic hydrocarbon structure absorption band; the 1000–1800 cm^-1^ band was an absorption band with oxygen-containing functional groups and partial aliphatic hydrocarbon structure; the 2800–3000 cm^-1^ band was an absorption band of aliphatic hydrocarbon structure; and the 3000–3600 cm^-1^ band was a hydroxyl absorption band [23].

**Table 4:** Infrared absorption peak parameters of lignite aromatic structure (700–900 cm^-1^ band).

Peak number	Peak position (cm^-1^)	Peak area	Proportion (%)	Functional group
1	704.97	0.0096	0.30	Benzene ring disubstituted (4H)
2	752.70	0.78	24.65	Phenyl trisubstitution (3H)
3	775.96	0.91	28.92	Phenyl trisubstitution (3H)
4	796.33	0.78	24.79	Phenyl trisubstitution (3H)
5	815.52	0.50	15.98	Tetrasubstitution of benzene ring (2H)
6	833.58	0.12	3.96	Tetrasubstitution of benzene ring (2H)
7	872.54	0.042	1.34	Benzene ring pentasubstitution (1H)
8	894.26	0.0020	0.064	Benzene ring pentasubstitution (1H)

**Table 5:** Infrared absorption peak parameters of oxygen-containing functional groups of lignite (1000–1800 cm^-1^ band).

Peak number	Peak position (cm^-1^)	Peak area	Proportion (%)	Functional group
1	1053.35	8.78	3.44	C-O vibration of phenol, alcohol, ether, and ester
2	1102.68	22.46	8.81	C-O vibration of phenol, alcohol, ether, and ester
3	1202.24	47.85	18.77	C-O vibration of phenol, alcohol, ether, and ester
4	1322.06	51.06	20.03	C-O vibration of phenol, alcohol, ether, and ester
5	1451.42	56.90	22.32	Deformation vibration of CH_3_‒ and CH_2_‒
6	1567.51	25.65	10.06	Vibration of aromatic hydrocarbon C = C skeleton
7	1620.10	20.68	8.11	Vibration of aromatic hydrocarbon C = C skeleton
8	1692.20	21.59	8.47	Flexural vibration of carboxylic acid C = O

**Table 6:** Infrared absorption peak parameters of lignite aliphatic hydrocarbons (2800–3000 cm^-1^ band).

Peak number	Peak position (cm^-1^)	Peak area	Proportion (%)	Functional group
1	2820.08	0.22	2.73	Telescopic vibration of symmetric CH_2_
2	2842.54	0.92	11.24	Telescopic vibration of symmetric CH_2_
3	2861.93	0.97	11.81	Telescopic vibration of symmetric CH_2_
4	2886.45	1.24	15.16	CH telescopic vibration
5	2909.38	1.48	18.03	Telescopic vibration of asymmetric CH_2_
6	2930.20	2.12	25.81	Telescopic vibration of asymmetric CH_2_
7	2955.26	0.94	11.50	Telescopic vibration of asymmetric CH_3_
8	2977.15	0.30	3.71	Telescopic vibration of asymmetric CH_3_

**Table 7:** Infrared absorption peak parameters of lignite hydroxyl group (3000–3600 cm^-1^ band).

Peak number	Peak position (cm^-1^)	Peak area	Proportion (%)	Functional group
1	3038.85	4.63	5.12	OH‒N hydrogen bond
2	3083.36	8.65	9.57	OH‒N hydrogen bond
3	3158.60	18.18	20.12	Cyclic hydrogen bonding
4	3238.42	11.30	12.50	Cyclic hydrogen bonding
5	3315.05	15.92	17.61	OH‒O hydrogen bond
6	3391.97	14.12	15.63	OH‒OH hydrogen bond
7	3462.09	12.63	13.98	OH‒π hydrogen bond
8	3532.23	4.94	5.47	OH‒π hydrogen bond

#### 3.2.1 Aromatic hydrocarbon structure

The fitting results showed that the main absorption peak of aromatic structure in Tiebei lignite was between 750 and 810 cm^-1^, which belonged to benzene ring trisubstitution. The peak area of benzene ring trisubstitution was 2.47, and the relative area ratio was 78.36%. The peak area of the tetrasubstituted benzene ring was 0.62, and the relative area ratio was 19.94%. The results showed that the main functional groups in the macromolecular structure of Tiebei lignite were trisubstituted benzene rings, which were supplemented by four or five substituted benzene rings, and the content of disubstituted benzene rings was relatively small. The quantitative analysis of different benzene ring substitution methods and quantities in the macromolecular structure of Tiebei lignite provided the theoretical basis to accurately construct the macromolecular structure of Tiebei lignite.

#### 3.2.2 Oxygen-containing functional groups

Oxygen-containing functional groups in coal included hydroxyl group (OH), carboxyl group (‒COOH), carbonyl group (C = O), and ether oxygen bond (R‒O‒R′). Other than the oxygen-containing functional groups, there were aromatic carbon-carbon double bonds and aliphatic hydrocarbons in the 1000–1800 cm^-1^ band. Among them, the absorption peak area caused by C-O vibration of phenol, alcohol, ether, and ester was 130.15, accounting for 51.05%. Both absorption peaks at 1567.51 cm^-1^ and 1620.10 cm^-1^ belonged to aromatic hydrocarbon C = C skeleton vibration, which accounted for 18.17%.

#### 3.2.3 Aliphatic hydrocarbon structure

The wave number was 2800–3000 cm^-1^, which belonged to the absorption range of ‒CH_x_ in lipid chain and lipid ring. The aliphatic hydrocarbon content of Tiebei lignite was mainly asymmetric CH_2_ stretching vibration, and the relative area ratio was 43.84%. The relative area ratio of symmetric CH_2_ was 25.78%, which showed that the fatty chain in the molecular structure of Tiebei lignite mainly had a short chain structure.

### 3.2.4 Hydroxyl groups

In the FT-IR spectrum, 3000–3600 cm^-1^ was in the absorption vibration region of hydroxyl, and the structural types of hydroxyl included the OH‒N hydrogen bond, cyclic hydrogen bond, OH ether hydrogen bond, OH‒OH hydrogen bond, and OH‒π hydrogen bond. The hydroxyl type of Tiebei lignite was mainly a cyclic hydrogen bond, with a peak area of 29.48 and a relative area ratio of 32.62%, followed by the OH‒π hydrogen bond and OH‒O hydrogen bond, with relative area ratios of 19.45% and 17.61%, respectively. The OH‒N hydrogen bonds and OH‒OH hydrogen bonds were the least, and the relative area ratios were 14.69% and 15.63%, respectively.

#### 3.2.5 Calculation of FT-IR structural parameters of Tiebei lignite

The molecular structure model of Tiebei lignite shown in Table 8 included the basic structural parameters of the Tiebei lignite, including the fatty chain length, aromaticity, condensation degree of aromatic ring, aromatization index, and aromatic carbon rate. The calculation formula is as follows [24].

**Table 8:** FT‒IR structural parameters of lignite.

Coal sample type	Fatty chain length	Aromaticity	Degree of condensation of aromatic rings	Aromatization index	Aromatic carbon rate
Lignite	2.90	0.38	0.068	5.66	0.66

The length of the aliphatic hydrocarbon chain revealed the length and branching degree of the aliphatic chain in the Tiebei lignite, which was expressed by the ratio of ‒CH_3_ to ‒CH_2_, as shown in formula (2). The larger the parameter, the longer the aliphatic chain of coal:

$$\frac{A_{1}(CH_{2})}{A_{1}(CH_{3})}=\frac{A_{1}(2900-2940{\;\text{cm}}^{-1})}{A_{1}(2940-3000{\;\text{cm}}^{-1})},$$ (2)

where A_1_ is the peak fitting area in the corresponding interval, dimensionless.

Aromaticity can describe the richness of aromatic functional groups to fatty functional groups, which can be expressed as follows:

$$I=\frac{A_{1}(700-900{\;\text{cm}}^{-1})}{A_{1}(2800-3000{\;\text{cm}}^{-1})},$$ (3)

The degree of condensation (DOC) of aromatic ring represents the DOC of the aromatic ring in Tiebei lignite structure, which is the ratio of the out-of-plane deformation vibration of aromatic ring CH in the area with a peak position of 700–900 cm^-1^ to the aromatic C = C skeleton vibration in the area with a peak position of 1600 cm^-1^, and it can be expressed as follows:

$$DOC=\frac{A_{1}(700-900{\;\text{cm}}^{-1})}{A_{1}(1600{\;\text{cm}}^{-1})},$$ (4)

The aromatization index (Har/Hal) is the ratio of the C = C vibration of aromatic hydrocarbons at the peak of Tiebei lignite from 1520 to 1650 cm^-1^ to the vibration of aliphatic hydrocarbons at the peak of Tiebei lignite from 2800 to 3000 cm^-1^, which can be expressed as follows:

$$\frac{H_{ar}}{H_{al}}=\frac{A_{1}(1520-1650{\;\text{cm}}^{-1})}{A_{1}(2800-3000{\;\text{cm}}^{-1})},$$ (5)

The aromatic carbon rate ($f_{ar}^{C}$) refers to the proportion of carbon atoms in the molecular structure of Tiebei lignite to the total carbon atoms, and the aromatic carbon rate of coal can be calculated as follows:

$$f_{ar}^{C}=1-\left[\frac{A_{1}(2800-3000){\;\text{cm}}^{-1}}{A_{1}(700-900){\;\text{cm}}^{-1}+A_{1}(2800-3000){\;\text{cm}}^{-1}}\times \frac{H}{C}\right]/\frac{H_{al}}{C_{al}},$$ (6)

where Hal/Cal is the ratio of H and C in the aliphatic group, which is 1.8.

### 3.3 XPS analysis

**Fig 7:**
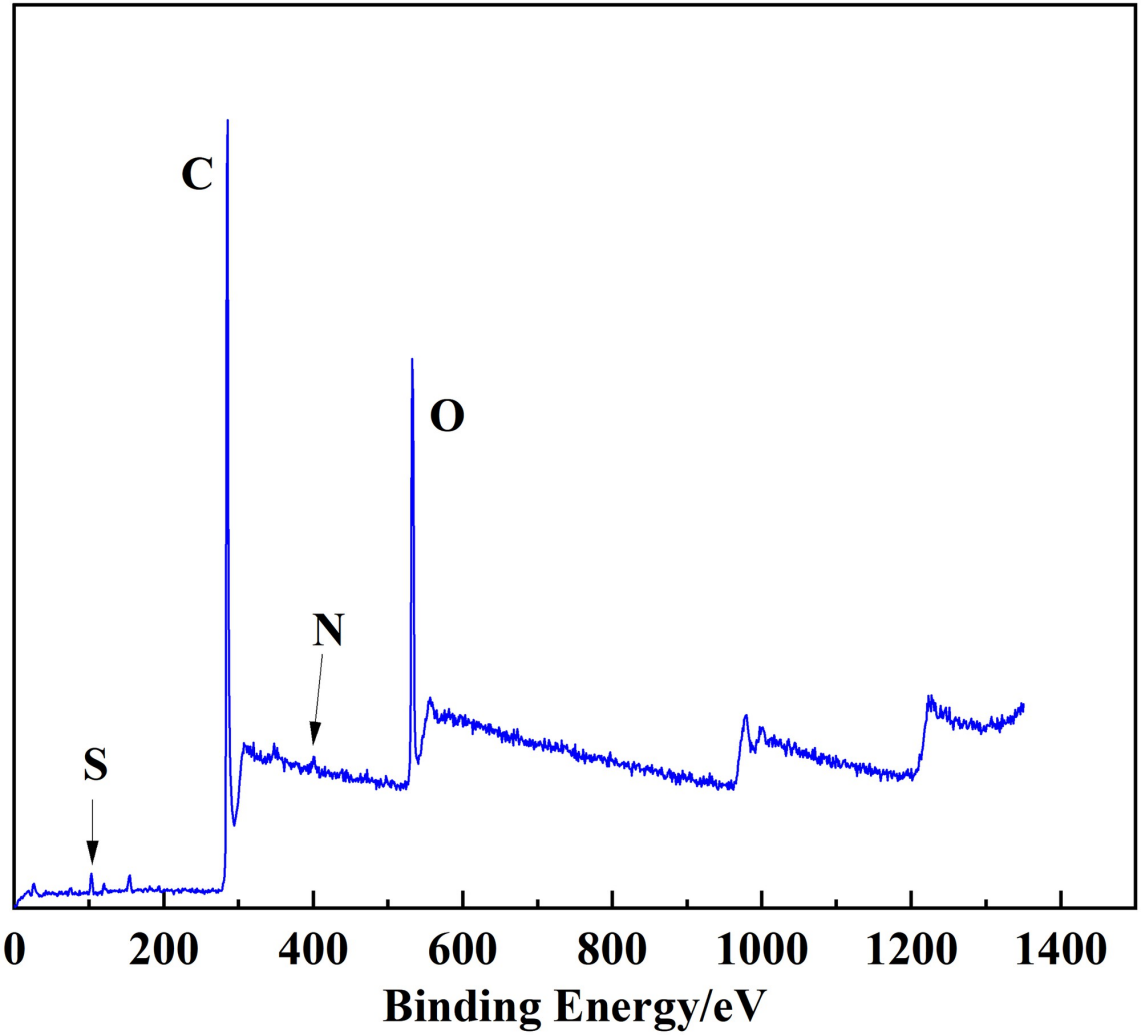
XPS spectrum of lignite.

**Fig 8:**
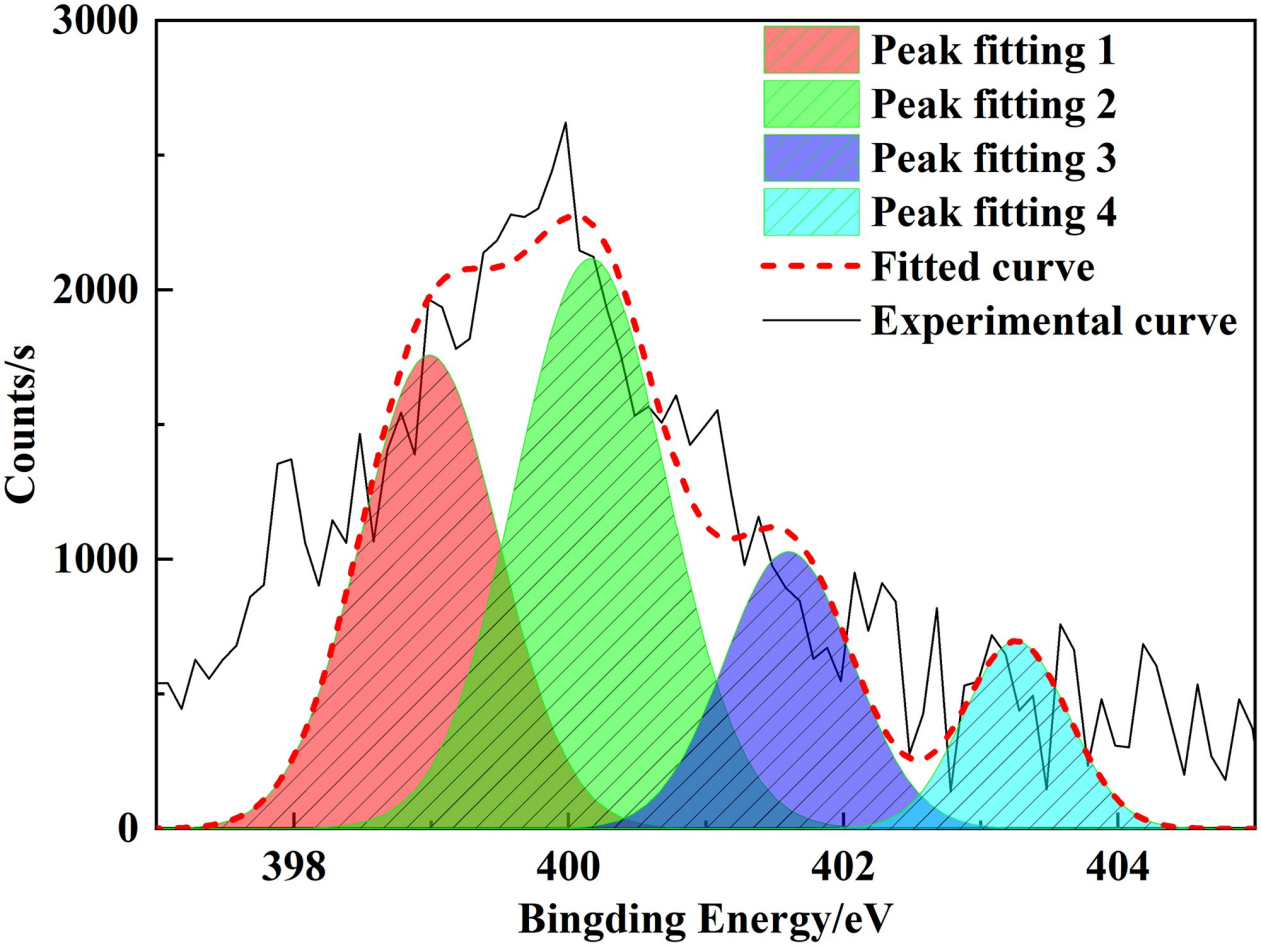
Peak fitting diagram of nitrogen in lignite.

**Fig 9:**
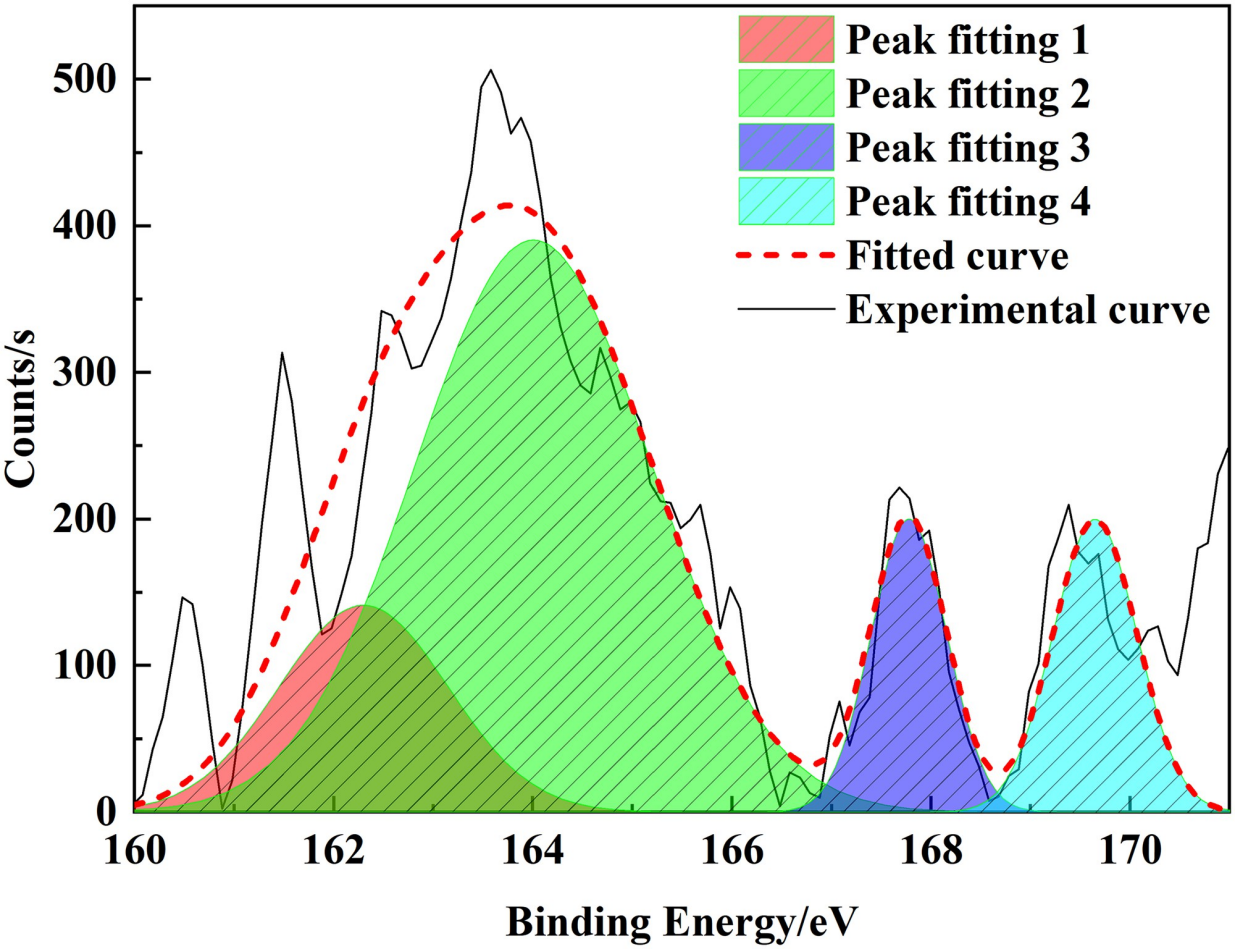
Peak fitting diagram of sulfur in lignite.

XPS is an analytical method that can be used to distinguish the existing state and relative content of different elements in coal [25]. The characteristic peaks of nitrogen and sulfur were fitted by peak splitting. In this study, we used XPS to determine the composition of heteroatoms in the Tiebei lignite molecule. Figs 7–9 show the XPS spectra and peak-fitting spectra of nitrogen and sulfur elements of Tiebei lignite, respectively. Table 9 presents the peak-fitting results of the existing forms and contents of nitrogen and sulfur elements to further clarify the occurrence characteristics of nitrogen and sulfur in Tiebei lignite.

**Table 9:** Existing forms and contents of nitrogen and sulfur elements in lignite.

Element	Belong to	Binding energy, *E* (eV)	Content, *w* (%)
Nitrogen element	Pyridine nitrogen (C_5_H_5_N)	398.99	31.88
	Pyrrole nitrogen (C_4_H_5_N)	400.16	41.57
	Quaternary nitrogen (‒N‒ (CH_3_) _3_)	401.60	17.10
	Nitric oxide (NxOy)	403.26	9.45
Sulfur element	Thiols and thioethers	162.30	16.09
	Thiophenes	164.01	62.29
	Sulfoxides	167.78	10.25
	Inorganic sulfur	169.65	11.38

(1) Occurrence form of nitrogen in coal samples

We obtained four peaks by fitting the XPS-N spectrum of coal samples: the peak was at 398.99 eV, which indicated that the structure belonged to pyridine nitrogen, accounting for 31.88%; the peak was at 400.16 eV, which indicated that the structure belonged to pyrrole nitrogen, accounting for 41.57%; the peak was at 401.60 eV, which indicated that the structure belonged to quaternary nitrogen, accounting for 17.10%; and the peak value was 403.26 eV, which indicated that the structure belonged to nitrogen oxide, accounting for 9.45%, which was formed by the oxidation of pyridine nitrogen and pyrrole nitrogen in air.

(2) Occurrence form of sulfur in coal samples

The main occurrence form of sulfur was thiophene, which accounted for about 62.29% of the total sulfur. Thiophenes had the characteristics of an aromatic structure. Thiols and thioethers accounted for about 16.09% of the total sulfur, whereas the amounts of sulfoxides and inorganic sulfur were relatively small, accounting for 10.25% and 11.38% of the total sulfur, respectively.

### 3.4 XRD analysis

**Fig 10:**
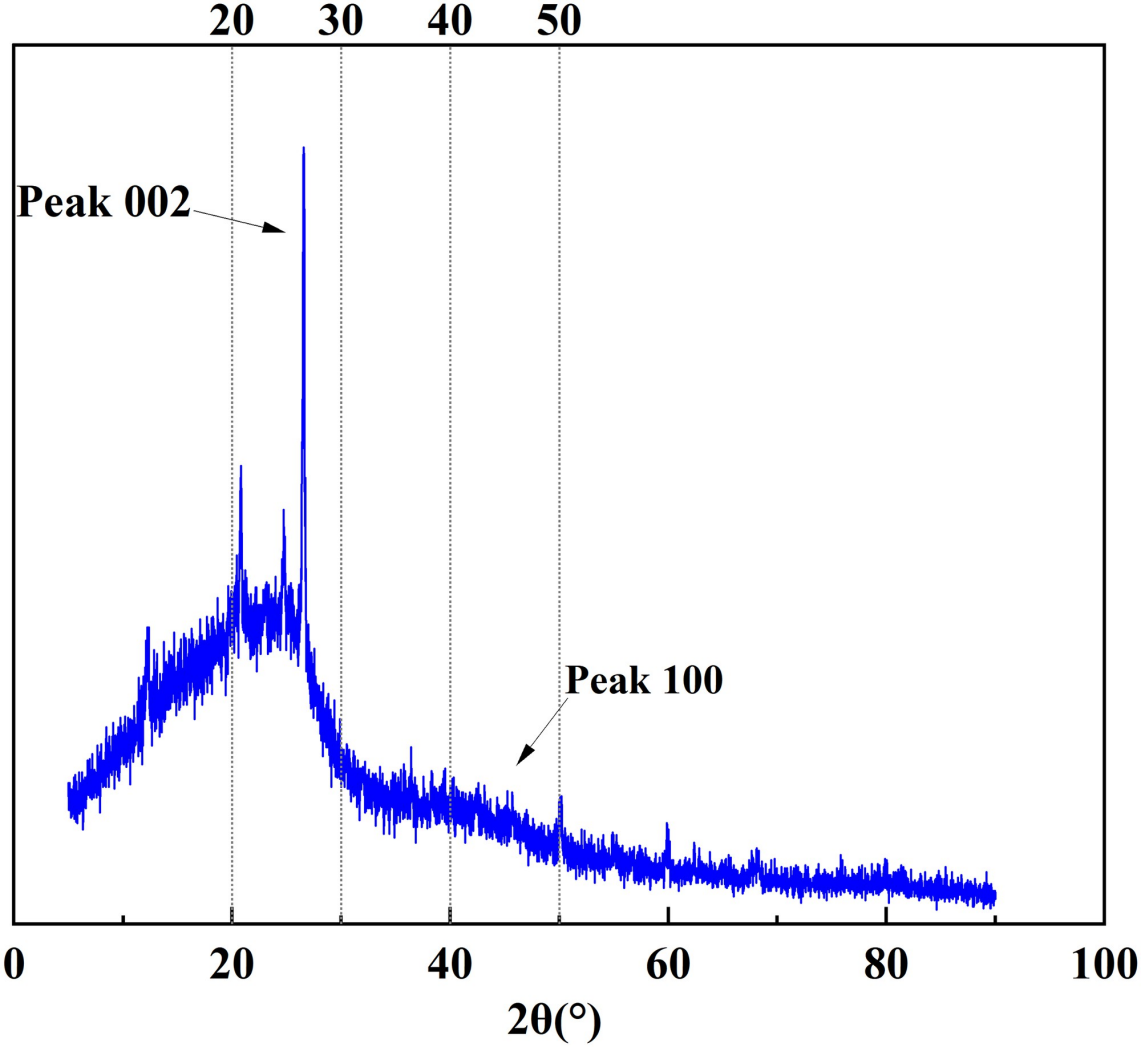
XRD spectrum of lignite.

**Fig 11:**
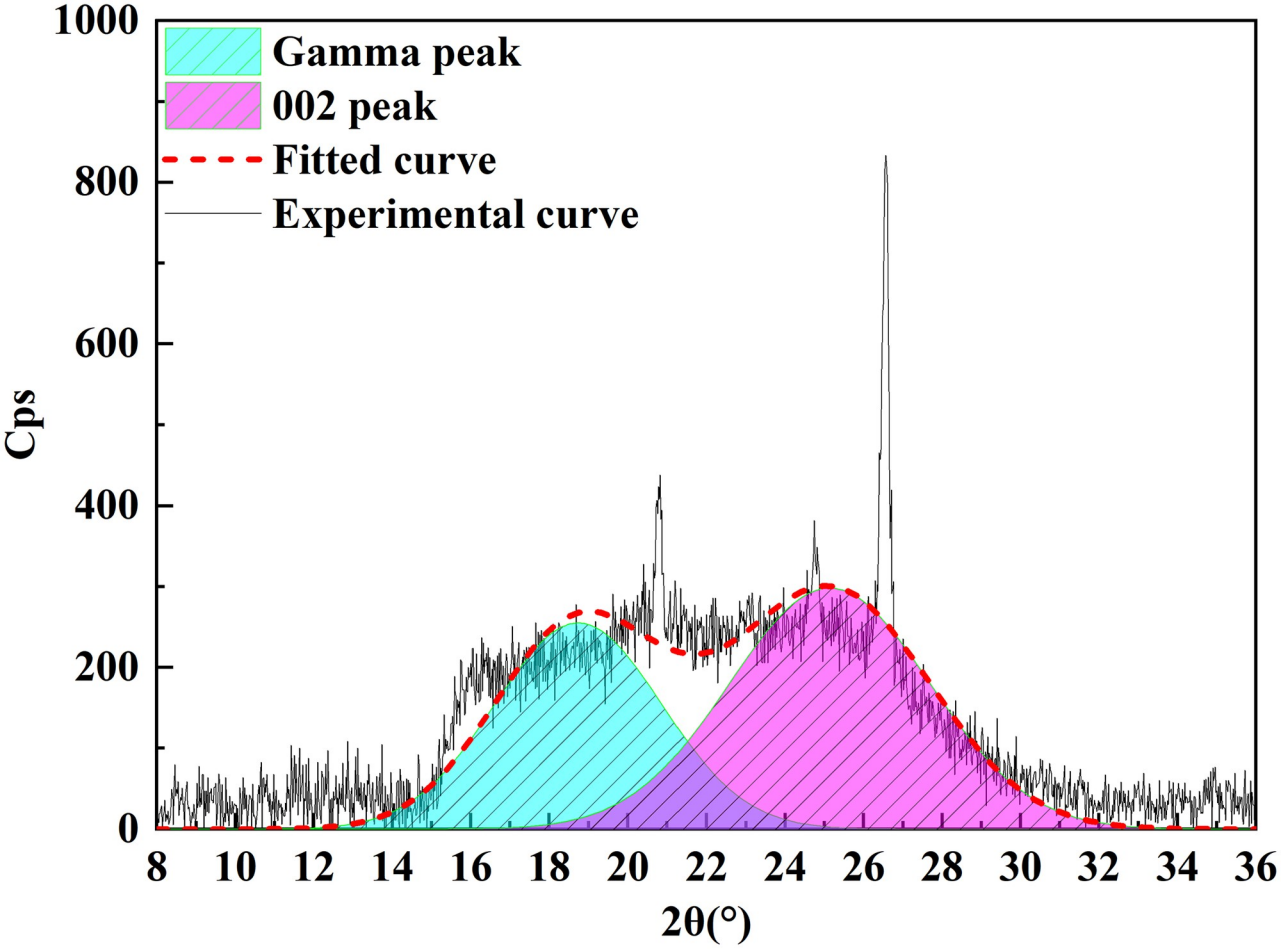
Peak fitting spectrum of lignite XRD‒002.

XRD analysis is a technical means used to study the structural characteristics of microcrystals, reveal the arrangement rules of carbon atoms, and characterize the structure of coal aggregation [26]. As shown in Fig 10, the XRD pattern of Tiebei lignite had two broad peaks at the diffraction angles of 2θ = 20°–30° and 2θ = 40°–50°, which were the 002 and 100 peaks of the microcrystalline structure, respectively. The 002 peak was formed by the superposition of the γ band and the 002 band (as shown in Fig 11), which reflected the spatial arrangement of the aromatic ring carbon and the distance between the aromatic ring carbon and aromatic ring layer. The γ band represented the fatty carbon structure in the Tiebei lignite, and 2θ = 40°–50° corresponded to the 100 peak of the microcrystalline structure, which indicated the condensation degree of the aromatic ring.

Using formula (7) [27] from Bragges and Scherrer, we calculated the interlayer spacing (d), ductility (L_a_), stacking degree (L_c_), stacking number (N_ave_), and aromaticity (f_a_) of Tiebei lignite. The calculation results of microcrystalline structure parameters of Tiebei lignite are shown in Table 10, which were the key parameters used to construct its molecular structure.

**Table 10:** Structural parameters of lignite microcrystals.

Coal sample type	*θ*_002_ (°)	*θ*_100_ (°)	*d*_002_ (nm)	*L*_*a*_ (nm)	*L*_*c*_ (nm)	*f*_*a*_	*N*_*ave*_
Lignite	12.57	21.57	0.354	2.395	1.416	0.584	4.004


$$\left\{\begin{array}{l} d_{002}=\frac{\lambda }{2\;\text{sin}\;\theta_{002}}\\ L_{a}=\frac{1.84\lambda }{\beta_{100}\;\text{cos}\;\theta_{100}}\\ L_{c}=\frac{0.94\lambda }{\beta_{002}\;\text{cos}\;\theta_{002}}\\ f_{a}=\frac{A_{002}}{A_{\gamma }+A_{002}}\\ N_{ave}=\frac{L_{\text{c}}}{d_{002}} \end{array}\right..$$ (7)

### 3.5 Molecular model of Tiebei lignite

#### 3.5.1. Construction of molecular model of Tiebei lignite

The ratio of bridge carbon to peripheral carbon of lignite was 0.14, which showed that the aromatic carbon structure in the model was mainly composed of benzene and naphthalene, supplemented by anthracene. Through a Matlab calculation [28], we obtained the types and quantities of aromatic structures closest to the bridge cycle ratio, as shown in Table 11. The total number of aromatic carbons in the model was 99. According to the ^13^C NMR analysis, aromatic carbons accounted for 51.98%, and so, the total number of carbons in the molecular structure of lignite was 190. According to the elemental analysis of coal samples, we determined that the carbon content of lignite was 58.93%, the oxygen content was 23.55%, the nitrogen content was 0.79%, and the sulfur content was 0.11%. We calculated that the structure had 57 oxygen and 2 nitrogen in the model. Because of the low sulfur content, the number was less than one, and so, the molecular structure of Tiebei lignite constructed in this study did not contain sulfur. According to XPS analysis, the main forms of N in lignite were pyridine nitrogen and pyrrole nitrogen, and the quantity ratio was about 1:1, and so, there was one pyridine nitrogen and one pyrrole nitrogen.

**Table 11:** Existing forms of aromatic carbon in the macromolecular configuration of lignite.

Existence form	Pyridine	Pyrrole	Benzene	Naphthalene	Anthracene
Quantity	1	1	6	4	1

**Fig 12:**
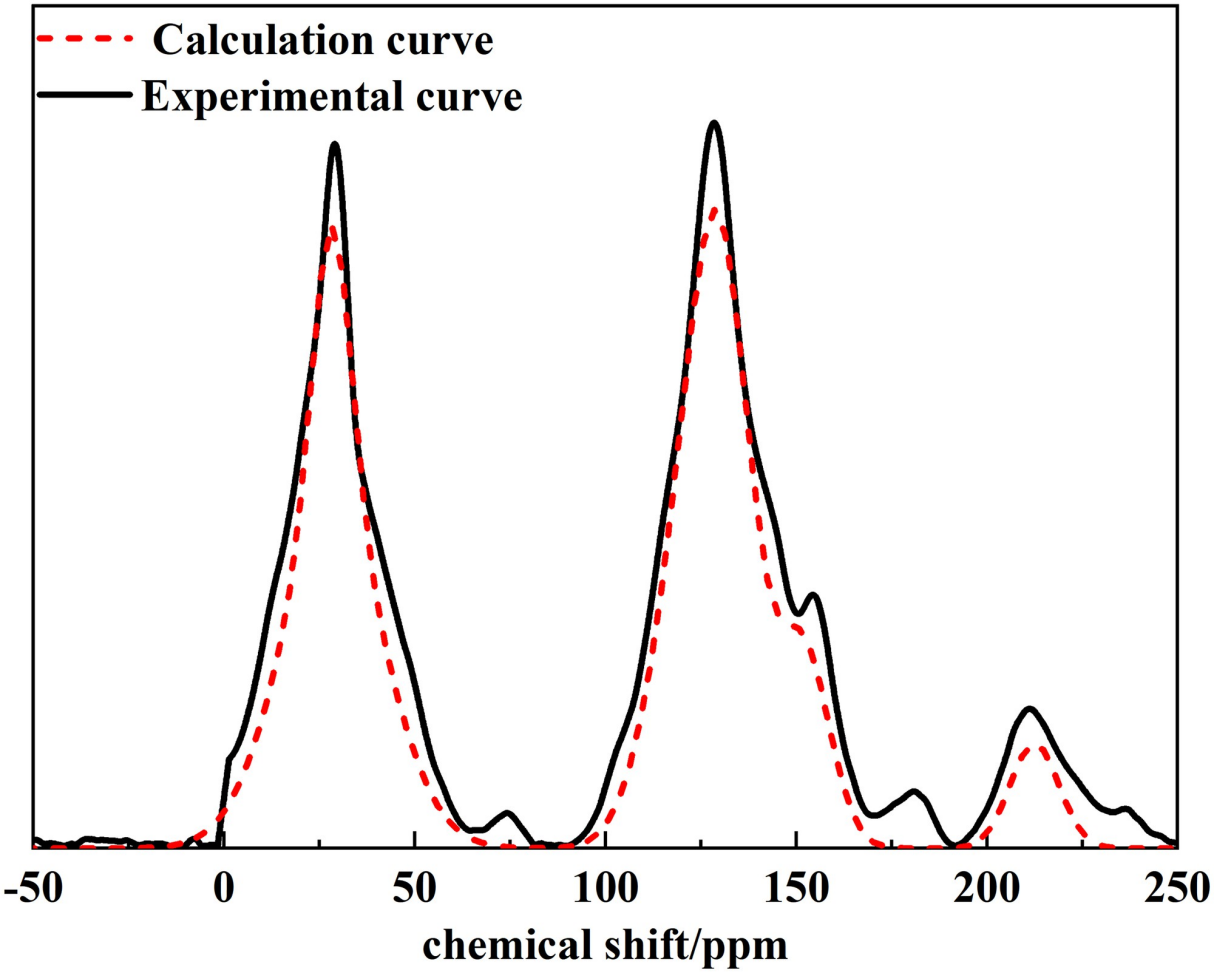
Comparison between ^13^C NMR experimental spectrogram and model calculation spectrogram of lignite.

**Fig 13:**
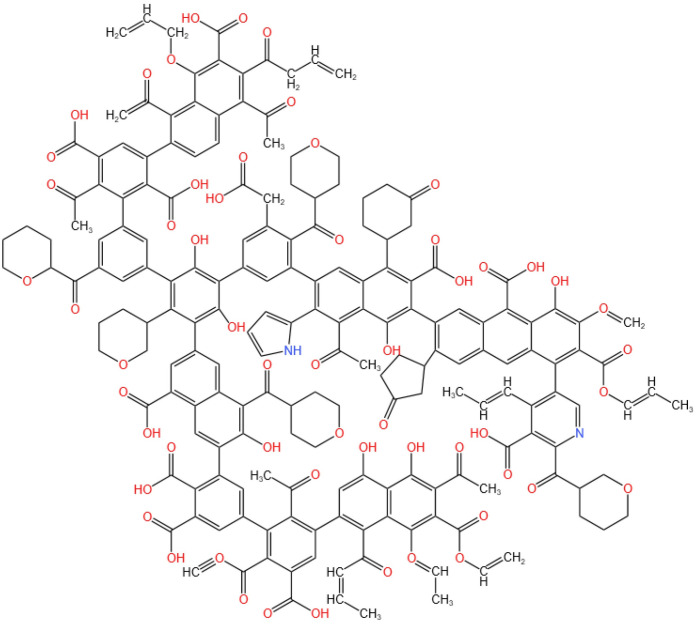
Plane model of the molecular structure of lignite.

According to the analysis results, we established the macromolecular model of Tiebei lignite, and then, we compared the ^13^C NMR spectrum of the model with the experimental ^13^C NMR spectrum, as shown in Fig 12. The results showed that the ^13^C NMR spectrum of the macromolecular model of Tiebei lignite established in this study was in good agreement with the experimental ^13^C NMR spectrum. Finally, we determined the molecular formula of lignite in the Tiebei Coal Mine to be C_190_H_161_O_57_N_2_. We drew a molecular structure model of Tiebei lignite using KingDraw software, as shown in Fig 13.

#### 3.5.2 Optimization of the molecular model of Tiebei lignite

**Fig 14:**
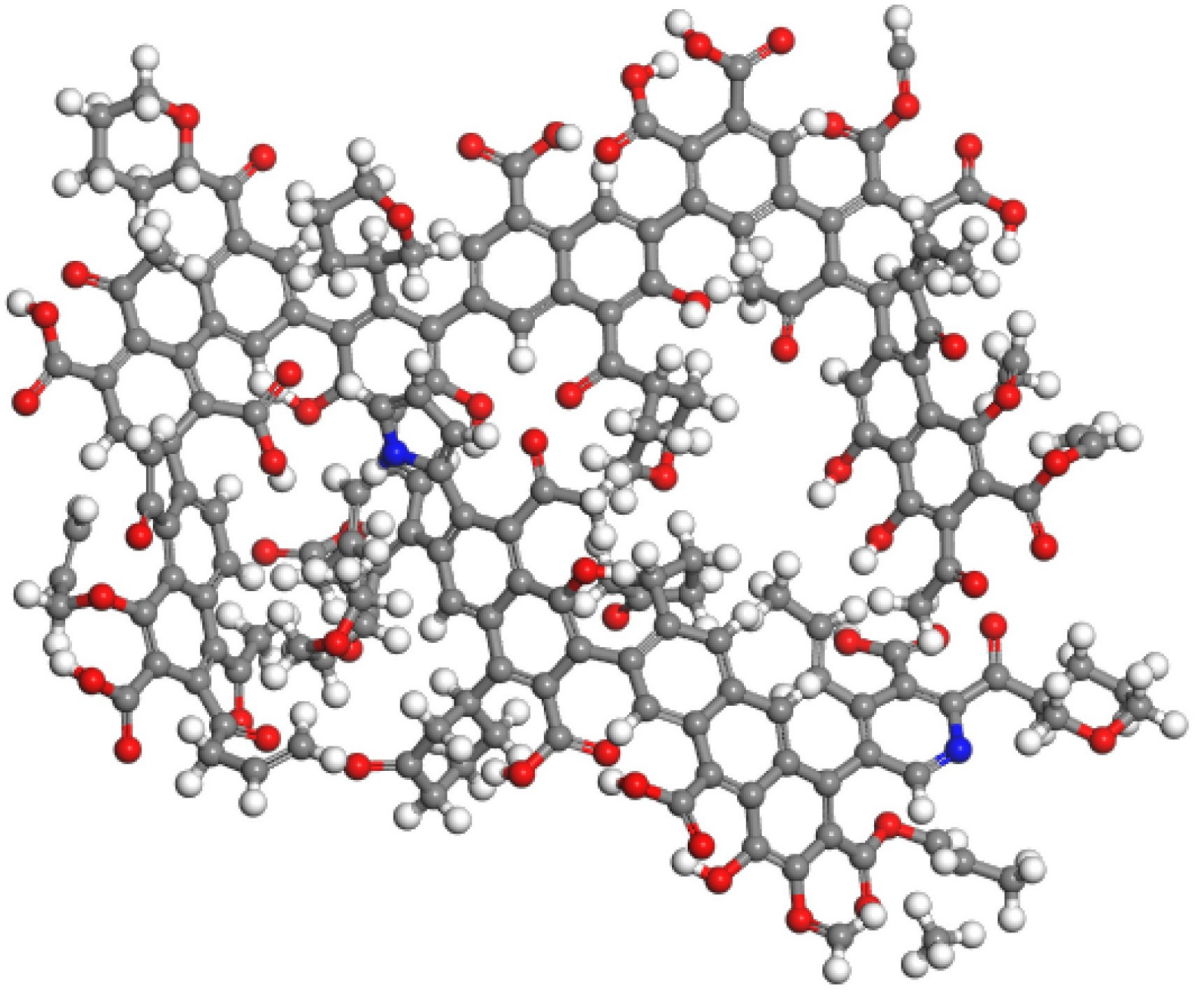
Optimized macromolecular structure model of lignite.

We introduced the two-dimensional plane molecular model of Tiebei lignite into the Materials Studio molecular simulation software [29]. We geometrically optimized the macromolecular structure of Tiebei lignite many times using the Forcite module, and the maximum number of iteration steps was 3000. The optimization method is Smart Minimizer method, For annealing and kinetic treatment, we selected the COMPASS forcefield, the calculation accuracy was set to Fine, and the Charges term was set to Forcefield assigned. We used the NVT dynamics ensemble, Choose Nose temperature control method; The initial temperature is set to 300 K, and the maximum temperature is set to 800 K; The heating rate is 60 K/time, and the simulation time is 1 fs; After each cycle, the output results are optimized by molecular mechanics again. and after several optimization treatments, we finally obtained the lowest energy configuration of the Tiebei lignite macromolecules, as shown in Fig 14. Based on the optimization of molecular mechanics and molecular dynamics, we found that some of the chemical bonds, such as bridge bonds and fatty bonds, were obviously twisted, which made the molecular structure model more stereoscopic and more practical. The initial total energy of the molecular structure before optimization was 39538.431 kJ/mol, the nonbonding energy was 2679.77 kJ/mol, and the valence electron energy was 12740.715 kJ/mol. After annealing optimization, the total energy of the molecular structure was 3577.233 kJ/mol, the nonbonding energy was 1593.642 kJ/mol, and the valence electron energy was 1983.585 kJ/mol. Because Tiebei lignite is a macromolecular structure composed of multiple aromatic rings, in the process of simulation, the molecular model of Tiebei lignite is transformed from a two-dimensional plane structure to a three-dimensional structure, and some chemical bonds in the molecular structure are twisted and the bond angle changes, resulting in an increase in bond angle energy and a decrease in bond expansion potential energy. As shown in Table 12, van der Waals energy was the main component of the nonbonding energy, and the energy value changed the most before and after optimization, which was the main factor that kept the molecular structure of Tiebei lignite stable.

**Table 12:** Energy of the macromolecular structure of lignite after optimization, kJ/mol.

Total energy		Nonbonding energy			Valence electron energy			
		Hydrogen bond energy	vander Waals energy	Coulomb energy	Bond expansion energy	Bond angular energy	Torsional energy	Inversion energy
Before optimization	39538.431	0.000	26845.364	−47.664	8843.335	243.320	3628.218	25.842
After the optimization	3577.233	0.000	1728.374	−134.732	413.368	499.347	1047.416	23.454

## 4. Conclusions

Based on the results of this study, we drew the following conclusions:

1. The molecular structure of Tiebei lignite was characterized by elemental analysis, FT-IR, XPS, XRD, and ^13^C NMR. The results showed that the ratio of aromatic bridgehead carbon to peripheral carbon X_bp_ was 0.14; the aromatic carbon rate was 51.98%; the aromatic carbon structure was mainly composed of benzene and naphthalene; the oxygen element existed in phenol, ether, carbonyl group, and carboxyl group; the nitrogen structure mainly existed in the form of pyrrole and pyridine; the sulfur element mainly existed in thiophene sulfur; and the aromatic hydrocarbon substitution was mainly in the form of trisubstitution.
2. According to the experimental analysis results, we drew the molecular structure model of Tiebei lignite using KingDraw software, and we determined the molecular formula of the molecular structure model of Tiebei lignite as C_190_H_161_O_57_N_2_. We optimized the macromolecular model of Tiebei lignite using the Materials Studio software, and the total energy of molecular structure after annealing optimization was 3577.233 kJ/mol. van der Waals energy was the main component of nonbonding energy, and the energy value changed the most before and after optimization, which was the main factor that kept the molecular structure stable.
3. The optimized macromolecular structure model of Tiebei lignite was more three-dimensional. This will help us to intuitively understand the molecular structure characteristics of Tiebei lignite and holds important guiding significance for the mechanism research of coal and gas outburst, coal spontaneous combustion, and other disasters.
